# Hospital and Institutional News

**Published:** 1914-10-31

**Authors:** 


### HOSPITAL AND INSTITUTIONAL NEWS.

### THE CUMBERLAND INFIRMARY, CARLISLE.

In the course of the controversy which has arisen on the imperfections of this infirmary, Sir Henry Burdett has referred to the disadvantages attaching to the absence of a local daily newspaper for Carlisle and the district around. Force is given to this point by an article which appeared in the *Carlisle Journal* of the 24th inst., which is in striking contrast to that in the *Cumberland News*, referred to last week, which treated judicially the important questions that have arisen. The first-mentioned article is neither good journalism nor fair criticism. Thus the *Carlisle Journal*, whilst refraining from publishing the original report and the further communications from Dr. Lediard and Sir Henry Burdett based upon it, contents itself with stating a case from its own point of view, or that of those who inspire its articles, which is as misleading as it is incomplete. *Carlisle Journal* readers are thus placed at a disadvantage, for they are kept in ignorance of the actual issues raised and their important bearing upon the efficiency of the Cumberland Infirmary and its administration. The *Carlisle Journal* declares that "The Hospital's suggestion that the Committee of Management is opposed to reforms in the system of management is devoid of truth and substance," but no such suggestion was made by us, and its parentage rests with our contemporary alone. Again the *Carlisle Journal* indulges in fiction when it refers to The Hospital's attempt to excuse Sir Henry Burdett's "inaccuracies on matters of fact," for we merely pointed out that these have been shown not to be inaccuracies of Sir Henry Burdett. We have reason to believe that the actual position at the infirmary during recent years demonstrates that the present system of administration should be thoroughly overhauled, a fact which should be within the knowledge of those who have it in their power to bring about an independent inquiry in the form suggested more than once in The Hospital. At any rate, the efficiency and position of this institution are now at the parting of the ways, and it rests with the governors to determine whether or not its administration shall be brought up to the standard of a modern hospital. Each city has the hospital that it deserves. Whether its standard of efficiency is high or low must rest with the citizens.

### DUTY-FREE RECTIFIED SPIRIT IN HOSPITALS.

We have referred on former occasions in The Hospital to the great help which it would be to the voluntary hospitals of Great Britain to be allowed the privilege of using duty-free rectified spirit in the making of tinctures, galenicals, &c., in the same way that duty-free industrial spirit is allowed in the making of lotions, liniments, the preservation of specimens, &c. The Inland Revenue authorities have been memorialised by various hospitals, but, we believe, to no purpose. In view, however, of the facilities which Governmental Departments have indicated their willingness to provide for the use of duty-free rectified spirit in the manufacture of commercial commodities, with a view to putting the English manufacturer and chemist on an equal footing with their Continental competitors, the present is an appropriate time for the British hospitals to join in an application to the Inland Revenue for the privilege. We would, therefore, suggest that the British Hospitals' Association should take the matter into their consideration, so as to secure the immediate co-operation of the principal hospitals, prior to a deputation being appointed to wait upon the authorities, or in such other way as may be deemed advantageous.

### THE VOLUNTARY HOSPITALS' CASE.

In this connection an institutional correspondent reminds us of one item upon which he suggests information should be fully obtained—namely, tincture of iodine, now so generally used in hospital wards and operating theatres for the sterilisation of the skin; and he submits that if duty-free rectified spirit could be used in its preparation hundreds of pounds would be saved to struggling institutions, and Government would thereby lend invaluable assistance to scientific research, which, carried out on a considerable scale on the Continent, is handicapped in England by the excessive duty, nearly 25s. per gallon. So we urge immediate application to the authorities of the Inland Revenue, because there has probably never been a time in the long history of the British voluntary hospitals when so much help has been rendered to the nation by hospital physicians and surgeons, hospital nurses, and hospital boards. Now that it is proposed to consider duty-free spirit in its relation to industrial enterprise in the British Isles, we are disposed to the view that the strong case which the hospitals can undoubtedly submit may receive careful consideration and be successful, subject, of course, to suitable arrangements being made to comply with conditions which may quite reasonably be laid down by the Customs and Excise Departments.

### AN ASSISTANT-SUPERINTENDENTSHIP GOING BEGGING.

Boards of Guardians are evidently beginning to realise that if they wish to obtain the services of capable medical assistants it will be necessary to revise the amount of salary given for these posts. The St. Pancras Guardians—despite several advertisements—have not received any applications from suitable persons for the post of senior assistant medical superintendent of the St. Pancras South Infirmary, and the infirmary committee now recommend that the salary be increased from £175 per annum to £225, with residence and board. This represents the second increase made in this salary during the present year, it having been raised about January last. It remains to be seen whether this will make any difference to the number of applications received. Certainly the amount now fixed cannot yet be regarded as adequate for the responsible duties devolving on the senior medical assistant at a large London infirmary. Especially is this so at the present time, when there are so many congenial posts open which promise more ready advancement than the Poor-Law medical service.

### RE-OPENING OF THE OUT-PATIENT DEPARTMENT AT CARDIFF.

As foreshadowed in our columns last week, the Cardiff public have now been officially informed that the out-patient department of King Edward VII.'s Hospital will be re-opened on and after November 2. The authorities have, however, found it necessary to restrict the service to fewer days than has been the case under normal conditions, and an attempt will be made to limit the number of ophthalmic cases on the lines indicated by the following notice: "Eye cases—Mondays and Thursdays, 1 p.m. to 1.30 p.m. The department will be open for all urgent eye cases. Refraction cases in school children will be excluded and only a limited number of spectacle cases in adults can be seen. To prevent disappointment, etc., the out-patient ticket, with the medical certificate filled in, must be sent to the secretary, at the hospital, in the first instance, who will then write stating on what day the patient should attend." Owing to the number of the staff serving on the staff of the 3rd Western General Hospital, Cardiff, or attached to units engaged on active service, expedient limitations as to attendance are thought likely to ensure greater efficiency and satisfaction for more serious cases than an out-patient department open every day of the week, with an irregular or unpunctual attendance of the staff.

### NEW TUBERCULOSIS DISPENSARY FOR THE CITY.

A tuberculosis dispensary for the City of London is now ready to start its work. Arrangements have been successfully carried out in conjunction with St. Bartholomew's Hospital for the institution of a tuberculosis department, which will serve the City on the lines now generally agreed upon in anti-tuberculosis dispensary work. In one respect, however, the City of London Tuberculosis Dispensary will differ from the ordinary borough or county dispensary—namely, in the circumstance that its work will relate not only to the strictly residential population (which is, of course, numerically small), but also to that very large population which daily enters the City intent on business. Some useful results may be confidently expected from the work of this dispensary, not the least important may be the locating of business houses in which considerable numbers of cases of "open" pulmonary tuberculosis are to be found. The dispensary itself is housed in St. Bartholomew's Hospital, and, no doubt, some opportunity will be afforded to the students of the hospital for making use of the clinical material without any interference with the work of the tuberculosis officer, and without offending the susceptibilities of individual patients.

### NEW FEATURES OF THE CARD-INDEX SYSTEM.

Two members of the staff of the hospital will exercise some supervision over the clinical aspect of the work, and Dr. Howarth, the medical officer of health for the City, will control the administrative side of the dispensary in his official capacity. The main work of the department will be left in the hands of Dr. Gibbins, M.D., D.P.H., who was formerly in charge of a similar dispensary at the Royal Hospital for Diseases of the Chest, City Road. We understand that an extended use has been made of the card-index system, and that several new features have been introduced on the cards and case sheets, which should make the records of the "Bart.'s" dispensary not only the most complete in London, but the most satisfactory for compiling useful records or for tracing and controlling patients. It may be hoped that some details of the working of this system will be made available for the information of other tuberculosis officers, in the light of practical experience.

### COLLECTIONS NOT AFFECTED BY THE WAR FUNDS.

Our correspondent from Cambridge writes that the support given by the various churches and chapels in Cambridgeshire and parts of Huntingdonshire, Bedfordshire, Northamptonshire, Norfolk, Suffolk, Essex, and Hertfordshire to Addenbrooke's Hospital, Cambridge, in the form of collections at their respective harvest thanksgiving services, shows a considerable increase on that of last year, which, considering the many new demands made upon the charitably disposed public, must be most encouraging to the committee of management of Addenbrooke's. This reflects great credit not only upon the clergy and ministers who have so ably pleaded the cause of the hospital, but upon those who have so generously responded to the appeal. We are given to understand that Mr. R. J. Coles, the superintendent, as in other years, about the end of July or early in August sent a special letter to every incumbent and minister in the very large district served by the hospital, pointing out the special needs of the institution, and emphasising the fact that the present time is one of great anxiety as to raising the additional £1,000 a year required efficiently to maintain the new building.

### INFIRMARIES, PRISONERS, AND SICK REFUGEES.

The St. Pancras Board of Guardians, at the request of the Local Government Board, has decided to hand over the St. Anne's Home for Aged Men at Streatham to the Government to be used as a receiving home for Belgian refugees. Provision has been made to receive at the Fulham Infirmary cases of sickness occurring amongst the refugees housed at the Earl's Court Exhibition. The test-house of the Nottingham Guardians is to be used for German prisoners. The Hammersmith Board of Guardians has allotted a small block at the infirmary on Wormwood Scrubs for the treatment of the sick or injured from the aviation and motor transport camps in the neighbourhood. The Darlington Guardians have arranged to receive a number of wounded soldiers in the infirmary, and over a hundred are now under treatment there.

### THE OPEN DOOR FOR NAVAL ORPHANS.

In accordance with time-honoured custom, on Trafalgar Day (October 21) the annual meeting of the Royal Naval and Marine Orphan Home, Portsmouth, was held at the Home, when the Commander-in-Chief, Admiral the Hon. Sir Hedworth Meux, G.C.B., K.C.V.O., presided. The report stated that there were 147 girls in the Home, and at the request of the Admiralty arrangements had been made to admit twenty-seven additional children when occasion arises. There are also thirty-six boys maintained by this institution in Swanley Orphan Home at the cost of nearly £800 a year. One of the best ways in which the nation can show its gratitude to the men of the Navy, who are fighting with courage and efficiency under the most trying conditions of warfare, is to make provision for their orphans. The standard of the Home is known to all who take an interest in these matters, and seems to be especially appreciated on the part of the men of the Fleet themselves, who last year, through the Trafalgar Day collection, contributed £1,778 to its funds. Clearly such an institution must not be allowed to suffer now that the sources of charity are shown to be true well-springs by the success of this year's many appeals. The war must of necessity make the Home more than ever an institution with an open door for children of the men for whom it was established.

### WHAT IS THE VALUE OF GIFTS IN KIND?

During the past few years collections of gifts in kind, such as fruit, vegetables, bread, eggs, butter, jam, meat, fowls, &c., have been organised in a number of villages around Cambridge for the benefit of Addenbrooke's Hospital. The advent of these collections was looked upon with considerable suspicion, and many were pessimistic enough to prophesy that they would not and could not continue. These collections, in spite of such pessimism, have proved a success in augmenting the funds very substantially. Our Cambridge correspondent writes that the usual village collection of gifts in kind has again been organised by Mr. H. P. Hodges, the schoolmaster of Isleham, a small town in the Fen District, and 3 tons 4 cwt. and 3 qrs. of fruit, vegetables, &c., were sent to Addenbrooke's quite recently, representing a contribution in value about £14 at least. This gift consisted of 2½ tons potatoes, ½ ton of apples, celery, onions, beet, carrots, cabbages, &c. There were, moreover, 14 lb. tea, 1 lb. coffee, 1 lb. cocoa, 22½ lb. jam, honey, &c., besides 7 lb. salt pork, 3½ lb. butter, two bottles pickles, two fowls, two towels, and 187 eggs. What better proof could be given of the value of such collections, when proper organisation is applied to them?

### HOW CHILDREN CAN HELP OUR HOSPITALS.

The children at the schools in a small county town in Cambridgeshire have for many years sent regularly each year between 900 and 1,000 new-laid eggs to Addenbrooke's Hospital, Cambridge, for the patients. Such gifts from the children have been greatly appreciated and financially useful. For the past two years the children have not been content with sending their usual gift of new-laid eggs, and have now voluntarily initiated among themselves a penny collection, which both last year and this year has amounted to one guinea. They are continuing this as an annual subscription to their county hospital. Interest every class and age individually in your hospital, and the subscriptions will begin to take care of themselves.

### DRUG CULTIVATION IN HOSPITAL GARDENS.

The Board of Agriculture and Fisheries has issued a pamphlet relative to the cultivation and collection of medicinal plants in England. It is pointed out that the main supply of leaves, roots, etc., for medicinal use has been obtained from mid-Europe—especially Germany and Austria. Foreign competition has ousted the British grower from the market. Now, however, the war has altered the position, and it would appear to be a favourable opportunity for the British grower to cultivate, at a living profit, drugs which have been up to now largely imported. Belladonna leaves and root are notably in demand, and the price has already risen 100 per cent., and it is pointed out that next year higher prices still will be realised, owing to the fact that at least two years is required to grow this in quantity. Up to now all supplies of belladonna (except a small quantity of root) have been obtained from the Continent. Chamomile, a herb chiefly cultivated in Belgium, should pay for growing. Dandelion also has been imported in quantity from Germany. Digitalis is fairly profuse, growing wild, but has only been cultivated on a moderate scale. It would seem that here we have a true opportunity to "make war on the enemy's trade," as the South of England is mentioned as particularly suitable for drug-growing and is also near the markets. The Board's pamphlet concludes: "In the ordinary way a few pounds of dried herb are only disposed of with difficulty, buyers requiring hundredweights or more. Next year, however, is likely to be the exception, and such quantities as can be obtained will be saleable. In any case it is best to get into touch with a buyer before commencing to grow or collect medicinal herbs. The drug-grower who has special facilities for dealing with these should be approached in the first instance." Is not this a chance of revenue for some of our country hospitals and institutions, where ground is not at present being utilised?

### THE REPORT ON PROPRIETARY MEDICINES.

A bulky Blue-book of 782 pages has now been issued which contains the minutes of evidence given before the Select Committee on Patent Medicines. Those who have the time and inclination to skim this remarkable document will find in it as strong a condemnation of the secret remedy traffic as that contained in the report of the Committee itself. The most convincing evidence against quack medicines was that tendered by some of the witnesses who presented the case for the proprietors of patent medicines, notwithstanding that an unanswerable case was made out by the witnesses on the other side. When the times are favourable to the making of new domestic legislation Parliament will be able to find in this Blue-book convincing arguments in support of a Bill to suppress a traffic which, in many of its aspects, is indistinguishable from fraud of a most revolting character.

### THE EFFICIENCY OF THE VOLUNTARY SYSTEM.

Some telling facts with reference to the efficiency of the voluntary hospital system, and incidentally to the administration of the Leicester Royal Infirmary, were brought forward at the recent quarterly meeting of the governors of that institution. Mention was made that the 5th Northern General Hospital was largely staffed from the medical and surgical staff of the Leicester Royal Infirmary; that six of the resident medical officers had been called up for military service; six of the certificated nurses appointed to Queen Alexandra's Imperial Military Nursing Service; others mobilised for service with the base hospital, and that an additional number might be called up at any time. Furthermore, in order to render the greatest service at a critical time, it had been decided to place a ward at the disposal of young men rejected for military service on account of physical disability. Mr. C. J. Bond, well known for his surgical work, had come forward from professional retirement to undertake the surgical treatment of these patients. In ways like this the voluntary hospital system is rendering vital service to the nation, and we are convinced that the efficiency of the Leicester Royal Infirmary and the voluntary hospitals generally will command adequate support from those who are always ready to encourage effective service by generous contributions.

### THE LATE MR. HOWARD COLLINS.

We are asked to state that the death certificate of the late Mr. Howard Collins records that the primary cause of death was mucous colitis, secondary cause toxæmia. We are glad to publish this official information, which we trust will meet the eye of everyone who read the obituary notice in our last issue.

### UNIVERSITIES AND THE SHORTAGE OF MEDICAL STUDENTS.

The very full reports which our Special Correspondent at Cambridge has supplied with reference to the war in relation to hospital activity in the university town will have prepared The Hospital's readers for the condition of things in regard to medical students now that the October term has well begun. Almost precisely 50 per cent. of resident members are away, and this reduction is fully represented in the small numbers of medical students. According to the University correspondent of the *Times*, the entry of medical students this year is 64, as compared with 116 last year, 110 in 1912, and 114 in 1911. The coming shortage of doctors thus indicated is certainly serious. We pointed out its dangerous prospect at the beginning of the war, and the General Medical Council is, no doubt, fully alive to it. Efforts have therefore been made, at those centres of education where there are faculties of medicine, to encourage intending medical students still to continue their studies, even if they are otherwise fitted to enlist. This, in many instances, has been successful, but, of course, it has only mitigated the problem, not in any sense solved it. At present the various palliative measures discussed in The Hospital of August 15, pp. 551, 552, are not likely to be enforced. But the problem itself is of great importance not only in its immediate bearings, but in relation to the effects on disease, surgery, public health, and statistics which may be occasioned by a shortage of medical men. In this connection a correspondent writes: "Any shortage of medical officers, naval and military, would be a national misfortune, and it might be considered advisable to make a medical career possible to a certain number of students by granting a State subsidy to defray the cost of their education, while retaining an option upon their future services." What are our readers' views?

### A DELICATE QUESTION OF DISCIPLINE.

In reference to the general question raised in our leading article we should like to refer to one matter in particular. Flirtation is a delicate question to which to allude, but it undoubtedly forms a stumbling-block with which the unwary easily comes in contact. The student, who in a teaching hospital was one of many, finds himself alone, or almost alone, amongst a large staff of nurses. With the slightest cause, or without any cause whatever, charges of flirtation or favouritism, even of suspicion of immorality, may arise and may become serious in the extreme. Medical men should be exceptionally well able to appreciate the danger of affording any opportunity for charges which may even arise in the case of the most innocent. The rule of the experienced hospital matron that no nurse engaged to be married to anyone working on the hospital staff should remain a member of the nursing staff is, although at first sight drastic and unsympathetic, a good rule founded on common sense.

### A HEDGE OF PETTY RESTRICTIONS.

There are certain matters connected with the personal conduct of the "resident" which are especially liable to give rise to difficulties, and concerning which committees and governing bodies have proved to be only too fond of making detailed, and even grandmotherly, regulations, to the annoyance and discomfort of those affected. When the liberty of the occupant of a resident post is found to be surrounded by a hedge of petty restrictions it may be taken as certain that these have arisen as a consequence of the peccadilloes or the thoughtlessness of some of those who have previously held it. The house surgeon or junior medical officer who is told that it is a rule that he must return from leave by ten o'clock, or that he must pay 1s. 6d. for the dinner of a visitor, may safely conclude that a predecessor has acted immoderately or indiscreetly. The junior doctor's quarters are not intended, and are not suited, as a common room for fellow-students, or to be used for a smoking-concert with choruses resounding during the early morning. What time can reasonably be regarded as leisure time varies widely in different circumstances; there is, however, nowadays always much laboratory and other work to be done, and modern methods of treatment entail the devotion of more time than was required to make a routine examination and write a prescription. The point is worth allusion, since residents are always changing, and one generation does not know that its restrictions have generally been imposed by the sins of its predecessors.

### THE EARLY-CLOSING DAY AT CHEMISTS' SHOPS.

The cases of hardship due to the closing of all chemists' shops in a large area are, fortunately, very rare, although there is little doubt that the public has suffered considerable inconvenience, especially since the passing of the National Insurance Act, which has made them so thoroughly dependent on the chemists for their supply of medicine. An instance of a painful character, however, has occurred at Brixton. Facts disclosed at a recent inquest before Mr. G. P. Wyatt, the Coroner, showed that a man was taken ill at his lodgings. His landlady went to the doctor, who gave her a prescription to take to the chemist. It was Wednesday, the early-closing day in the district, and she found the shop shut. A visit to a second shop also found that closed, a fact which drew from the Coroner the remark, "So in the meantime the man must die." Finally, she had to return to the doctor, who made up a bottle of medicine himself. The man died the same day of shock from peritonitis. The Coroner said he considered it absurd to shut up all the chemists' shops on the same day at the same time. He supposed these regulations were made by people who knew nothing about it. Now the idea was to close all shops at the same time for a half-day in London, which was ridiculous in the extreme. The Coroner's remarks are to a certain extent justified. The National Insurance Act has to a great extent made the medical profession dependent on the chemist and druggist for the dispensing of his prescriptions, and consequently he does not stock the same amount and variety of drugs that he did before the passing of the Act. Some arrangement certainly should be made whereby at least one chemists' shop could remain open in a large district for the convenience of the sick.

### RADIOLOGIST AT CRUMPSALL POOR-LAW INFIRMARY.

The necessity for the appointment of specialists at our large Poor-Law infirmaries is being realised slowly, but very slowly, by Boards of Guardians. The latest to make a move in this direction are the Manchester Guardians. They have just appointed Dr. Green as radiologist to the Crumpsall Infirmary at a salary of £50 per annum. The workhouse and infirmary have accommodation for about 3,500 inmates, so that there will be no lack of work. The infirmary already possesses a visiting surgeon, a visiting physician, and a visiting obstetrician. It labours under the disadvantage, however, of being still unseparated from the workhouse, and does not possess a resident medical superintendent, so that there can be no proper co-ordination of the medical work.

### AN ENGLISH DOCTOR WITH THE GERMAN WOUNDED.

Dr. Barty King will give a lecture, illustrated by the epidiascope, on "Germany in War Time," with an account of his experiences among the German wounded, to the nurses of the Tuberculosis School of the Royal Hospital for Diseases of the Chest, City Road, in the lecture theatre, on Tuesday, November 3.

### THE LACK OF MENTAL HOSPITAL BEDS.

According to a report of the London Mental Hospitals Committee, applications for the admission of recent cases of certified lunacy continue to be received in large numbers, the applications from the beginning of the year to September 26 amounting to 3,076, and showing an increase of 290 as compared with the applications for the corresponding period of the previous year, and 209 as compared with the corresponding period of 1912. Recently it has not been possible, except after considerable delay, to deal with all the applications for beds, owing to there not being sufficient vacant beds in the London mental hospitals. At present ninety-two male and seventy-nine female patients are awaiting removal from out-county and borough mental hospitals and licensed houses, and the early transfer to London mental hospitals of some of these cases is being pressed for. The authorities of the Surrey County Mental Hospital and the East Sussex Mental Hospital have given notice to terminate their existing contracts for the reception of thirty-five male and thirty female patients, but a further contract has been entered into with the Essex County Mental Hospital at Brentwood for the reception of twenty-five female London patients. These figures show as clearly as possible the need for the new mental hospital which the County Council is now building. They are also interesting in reference to the fact, discussed elsewhere, that since the war a number of persons have been precipitated into mental disease, and have sought treatment in private and public mental hospitals.

### THE SALARIES OF MENTAL HOSPITAL STAFFS.

Dr. C. E. Hetherington, resident medical superintendent of the Derry District Mental Hospital, Ireland, has recently requested the committee of management to consider the question of raising the salaries of the medical staff, particularly that of the junior medical officer. At a subsequent meeting, in discussing this proposal, Mr. H. T. Barrie, M.P., chairman, remarked that the remuneration of medical men had been raised since the passing of the Insurance Act, and again since the outbreak of war, owing to the scarcity of applicants for appointments. Additional remuneration, he added, was the only means of attracting young men, and they would have to make up their minds to raise the salary of the junior medical officer from £150 to £200 a year. Apparently the committee were not disposed to make up their minds too promptly to this course, for after discussion it was agreed to advertise the post at £175 per annum. We venture to think that the adoption of Mr. Barrie's suggestion would have proved a quicker way of filling the appointment; for at the present time the salary of £175 is not too attractive for a junior medical post in a mental hospital, especially at the present day when the whole conditions of the mental hospital service are being canvassed and wide discontent among the junior men is being expressed. Whether first or last, therefore, we believe that the committee of the Derry District Institution will have to raise their projected salary to £200 before a suitable candidate is found.

### INFIRMARIES AND SCHEMES OF WORK.

Efforts are being made to cope with the acute distress in London due to the war, and the Camberwell Board of Guardians have decided to help unemployed painters and builders in the borough, as suggested in The Hospital of September 5, page 615. So they have discharged all inmate labour employed on painting their infirmary, and, shortened the hours of the men to thirty-six a week, and decided to pay them 9½d. an hour. By this means work will be found for double the number of men. They have also decided to take in hand the painting of the interior of the nurses' block, which in the ordinary course of events would not be done until next year. They will not advertise for tenders, but have the work done by the genuine unemployed under the supervision of the hospital staff. They have again resolved to ask the Local Government Board to sanction the erection of a separate operating-theatre for septic cases. When this question was placed before the Local Government Board some eighteen months ago, they refused their sanction, but with a new head of the department there may be an alteration of opinion as to its utility. Objection was raised to the scheme at the meeting of the Guardians, but Dr. Capes pointed out that there was not a hospital with 800 beds in the metropolis without a separate operating-theatre for septic cases. It would be much better for the patients and the staff. The proof of this contention was contained in the special articles devoted to this subject which appeared in The Hospital of May 31, 1913 (p. 279), and June 21, 1913 (p. 361).

### A NEW TARIFF FOR DRUGS AND APPLIANCES.

Panel chemists have just had a very disagreeable surprise in the nature of an intimation conveyed to the Insurance Committees through the Commissioners that lower fees are to be allowed for dispensing in certain cases. The essential facts are these: A new tariff for drugs and appliances which had been drawn up after consultation with the proper authorities was on the point of being agreed to by the Insurance Committees, when at the last moment these Committees received a circular-letter from the Commissioners to the effect that they would require the insertion of the following clause in the new tariff before giving their approval to it: "If a formulary or special Pharmacopœia is adopted locally for use in prescribing by practitioners on the panel, dispensing fees will be charged at half the rates set forth above in respect of such of the mixtures specified therein, not exceeding ten at any one time, as may from time to time be selected for the purpose by the Panel Committee, the reduced rates to take effect as regards any mixtures so selected one calendar month after notice of the intention to apply those rates thereto has been issued by the Insurance Committee to persons supplying drugs and appliances. Provided that the reduced rates will only apply to those mixtures which have been agreed by the Panel Committee and the Pharmaceutical Committee, or, failing agreement between them, adjudged by the Commissioners to be capable of being stocked in bulk without deterioration. The application of the reduced rates to any particular mixture is liable to be terminated by the Panel Committee, with the consent of the Insurance Committee, on notice being issued by the Insurance Committee to persons supplying drugs and appliances, as from the date specified in such notices." The effect of this clause will be dealt with in our Notes next week.

### THIS WEEK'S DRUG MARKET.

There appear to have been no abnormal fluctuations in values during the week, and the market has settled down more or less comfortably to the new conditions. Buying is restricted to a very small scale, which is only natural in view of the new standard of prices; but the demand is small, even in the case of drugs, of which the prices have not materially advanced. Cod-liver oil, for instance, is a very quiet market at a figure which can only be regarded as remarkably low considering all the circumstances. Buyers could not go very far wrong, one would think, in securing a sufficient supply of this article to last through the winter, for while there is some probability that prices will advance considerably the chances of a reduction appear to be small. Chamomiles are again dearer. The rapid advance in the quotations for potassium permanganate has received a check, and prices are, in fact, lower in consequence of the arrival of supplies from neutral ports. As was anticipated, carbolic acid is again dearer. Opium and morphine are practically unchanged, but the price of codeine has an upward tendency.

### TO OUR READERS.

Contributions are specially invited from any of our readers to these columns. They should deal with topical subjects and news. They must be authenticated for the information of the Editor only. The minimum payment if published is 5s. There is no hard-and-fast rule as to space, but notes of about twenty lines in length are preferred.

